# Absolute Rheological Measurements of Model Suspensions: Influence and Correction of Wall Slip Prevention Measures

**DOI:** 10.3390/ma13020467

**Published:** 2020-01-18

**Authors:** Sebastian Pawelczyk, Marieluise Kniepkamp, Steffen Jesinghausen, Hans-Joachim Schmid

**Affiliations:** Particle Technology Group, Paderborn University, 33098 Paderborn, Germany; sebastian.pawelczyk@upb.de (S.P.); marie.kniepkamp@googlemail.com (M.K.); hans-joachim.schmid@upb.de (H.-J.S.)

**Keywords:** wall slip prevention, effective gap height, parallel-plate system, structured surfaces, model suspensions, cement paste, fresh concrete

## Abstract

Since suspensions (e.g., in food, cement, or cosmetics industries) tend to show wall slip, the application of structured measuring surfaces in rheometers is widespread. Usually, for parallel-plate geometries, the tip-to-tip distance is used for calculation of absolute rheological values, which implies that there is no flow behind this distance. However, several studies show that this is not true. Therefore, the measuring gap needs to be corrected by adding the effective gap extension δ to the prescribed gap height H in order to obtain absolute rheological properties. In this paper, we determine the effective gap extension δ for different structures and fluids (Newtonian, shear thinning, and model suspensions that can be adjusted to the behavior of real fluids) and compare the corrected values to reference data. We observe that for Newtonian fluids a gap- and material-independent correction function can be derived for every measuring system, which is also applicable to suspensions, but not to shear thinning fluids. Since this relation appears to be mainly dependent on the characteristics of flow behaviour, we show that the calibration of structured measuring systems is possible with Newtonian fluids and then can be transferred to suspensions up to a certain particle content.

## 1. Introduction

**Figure 1:**
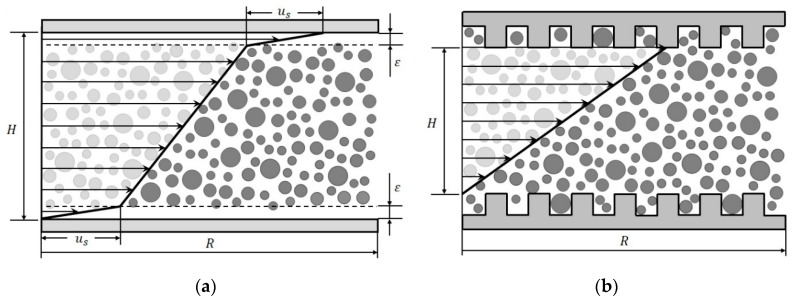
Schematic illustration of the velocity profile in a parallel-plate system (gap height H, plate radius R); (**a**) showing wall slip (slip velocity $u_{S}$) with the particle-depleted area ε; (**b**) with grooved surface to prevent wall slip.

Suspensions are nowadays used in numerous industrial areas (e.g., in food, cosmetics, and construction industries). However, their absolute rheological properties are very difficult to determine, which, for example, results from colloidal forces and particle interactions [1,2,3]. A very important suspension in construction industries is fresh concrete. Although concrete is one of the most used building materials, its exact rheological properties in the fresh state are difficult to determine, even today. This mainly is due to the complex components of concrete, which not only lead to time-dependent but also to stress-dependent behaviour [4,5]. In the past, fresh concrete was analysed with different kinds of so-called concrete rheometers [6,7,8], and many investigations on its rheological properties have been conducted, but no general solution was found [6,8,9]. The goal was and still is the exact determination of fresh concrete rheology, since this is crucial for developing predictive flow simulations, particularly for high-performance concrete. One of the most fundamental difficulties in characterizing suspensions is apparent wall slip, since it not only affects rheological properties such as yield stress [2,3], but also complicates the processing and manufacturing of suspensions [10]. Wall slip, which will be used in the following as a synonym for apparent wall slip, is characterized by a velocity jump or rather a sharply increasing velocity near the wall. In general, the reason for wall slip in suspensions is the development of a particle-depleted area ε in proximity to the wall, which has a much lower viscosity than the bulk material, see Figure 1a [11,12].

The low particle concentration in this near-wall region is caused by different phenomena, but one of the most important aspects is the geometric restriction between the particles and the wall itself. Since the particles cannot penetrate the wall, the minimum distance between a particle centre and a flat wall is the particle radius. Combined with shear induced particle migration away from the wall, the development of a particle-depleted area and, thus, wall slip can hardly be prevented [13]. Therefore, seen macroscopically, there is no wall adhesion, and absolute rheological properties cannot be determined. Typically, the size of the depleted layer is at least on the order of the particle radius [14].

In principle, wall slip can either be corrected or inhibited for absolute measurements. However, correction methods require a huge measuring effort and sometimes even more than one set of measuring equipment [15,16,17]. Thus, it is more common to modify the smooth surface of the measuring systems to inhibit wall slip. By roughening the surface or even structuring it, the geometric restriction is annulated, and the build-up of a particle-depleted area is prevented, see Figure 1b. In case of structured surface geometries, the tip-to-tip distance H between two surfaces (see Figure 1b) is usually used for further calculation of rheological properties, assuming that there is no flow behind it. However, different investigations showed that these assumptions are void.

Nickerson et al. [18] conducted research on a gap boundary extension $\delta^{\prime }$ with a cleated parallel-plate system. Investigating inter alia silicon oils and PDMS (poly(dimethylsiloxane)) putty, they discovered that the gap boundary for their cleated geometry has a value of $\delta^{\prime }=157\;\mu m$, which was in good agreement with their porous medium prediction, originally presented by Beaver and Joseph [19], of $\delta^{\prime }\sim 160\;\mu m$. Since the correction factor δ was applicable to both materials, they stated that δ is independent of material properties for a wide range of soft materials and fluids. Furthermore, they claimed that this gap boundary is independent of the length L of their cleated tools, since the obtained results were insensitive to their used cleat lengths of 0.6 mm and 1.3 mm. Similar investigations have been performed by Ferraris et al. [20], who analysed the influence of zeroing and serration on the measurement of calibration oils. Using a parallel-plate system as well, they determined a gap correction of $2\cdot \delta^{\prime }=270\;\mu m$ for serrated upper and lower plates with a structure depth L=381 μm. Since this value deviates from the results obtained by Nickersonet et al, the independency of δ from the structural depth might not be given and should be investigated in more depth. Another analysis was conducted by Carotenuto et al. [21], who glued sandpaper with different roughness on smooth plates. By investigating Newtonian fluids with smooth and rough parallel-plate systems, they proved that the material flowed indeed within the roughness. Variating the plate distance, they found a behaviour that resembles wall slip for the roughened plates, which was absent for smooth plates. This was attributed to the penetration depth. Furthermore, they described that the gap boundary δ not only changed with different roughness but was also dependent on whether only the lower plate was roughened or both.

As previous investigations show, the application of structured geometries leads to an increase in the effective gap size and, therefore, strongly influences the determination of viscosity values to an unknown extent. Nevertheless, structured geometries are widely used in commercial and especially in concrete rheometers to inhibit wall slip. Since there is a huge lack of information on how different geometries influence the rheological characterization of materials, we conducted research on three types of structured walls (pyramids, columns, and longwise bars) in a parallel-plate rheometer. The materials investigated in this study were chosen with the aim to predict the behaviour of complex fluids from the results obtained for Newtonian fluids. Besides Newtonian fluids, the flow of fresh concrete should be imitated by a shear thinning fluid and different suspensions. In any case, the measured viscosities were corrected by comparing them to reference values and determining the effective gap size. For every system, the effective gap size could be determined, which is crucial for obtaining correct rheological properties for such geometries.

## 2. Theory

Parallel-plate measuring systems consist of two parallel plates with radius R at a distance H aligned at the same symmetry axis. Typically, only the upper plate rotates [22]. Due to increasing circumferential speed from the centre of the plate to the edge, the shear rate $\dot{\gamma }$ increases likewise with rotational speed n and the angular velocity ω, see Equation (1) [22,23]:

(1) $$\dot{\gamma }\left(r\right)=\;\frac{\omega \cdot r}{H}=\frac{2\cdot \pi \cdot n\cdot r}{H}.$$

Since the calculation of a single shear rate is necessary for further investigations, we chose the maximum shear rate occurring at the edge of the plate (r=R):

(2) $${\dot{\gamma }}_{max}={\dot{\gamma }}_{R}=\frac{\omega \cdot R}{H}.$$

Another approach is the calculation of the shear rate at R=2/3, which makes the correction of shear stress dispensable for numerous materials [22]. Since this correlation is based on empirical investigations only, and the range of validity is unknown, particularly for structured plates, it might lead to misinterpretations for complex fluids.

The Weißenberg–Rabinowitsch correction for the shear stress at R can be derived mathematically and is more suited for scientific investigations. For a Newtonian fluid, the shear stress $\tau_{R}$ at the radius R is directly given in terms of the torque M:

(3) $$\tau_{R,N}=\frac{2\cdot M}{\pi \cdot R^{3}}.$$

For unknown flow behaviour, using Equation (3) for evaluating $\tau_{R}$ leads to erroneous results, which require correction. The value $\tau_{R,N}$ derived from Equation (3) is therefore corrected using this Weißenberg–Rabinowitsch correction [24]:

(4) $$\tau_{R}=\frac{M}{2\cdot \pi \cdot R^{3}}\cdot \left(3+\frac{dlnM}{dln{\dot{\gamma }}_{R}}\right)=\frac{\tau_{R,N}}{4}\cdot \left(3+s\right).$$

Following, the viscosity is calculated according to Equation (5):

(5) $$\eta_{R}=\frac{\tau_{R}}{{\dot{\gamma }}_{R}}=\frac{\left(3+s\right)}{4}\cdot \frac{\tau_{R,N}}{{\dot{\gamma }}_{R}}=\frac{\left(3+s\right)}{4}\cdot \frac{2\cdot H\cdot M}{\pi \cdot \omega \cdot R^{4}}$$

### Wall Slip Correction

**Figure 2:**
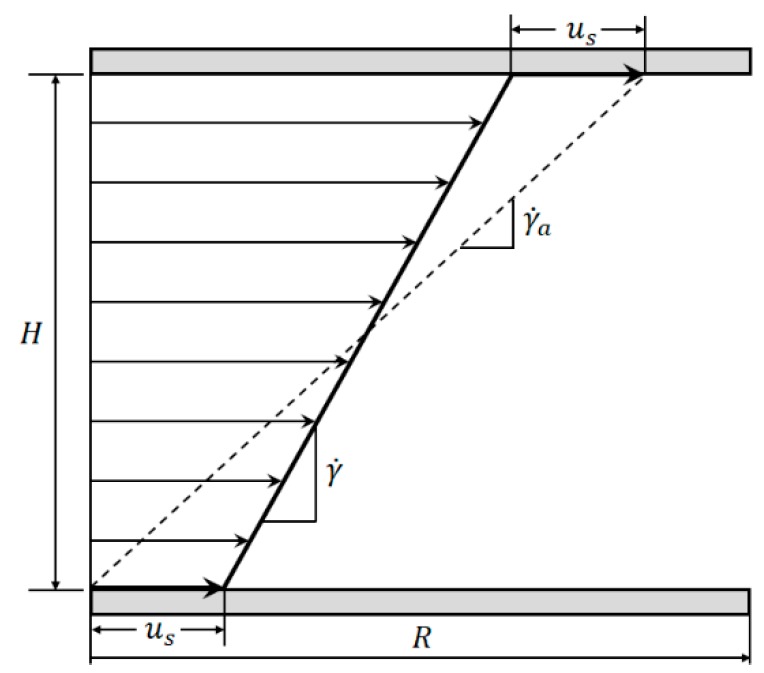
Comparison between apparent shear rate ${\dot{\gamma }}_{a}$ and true shear rate $\dot{\gamma }$ in the velocity profile between two parallel plates showing wall slip.

Measuring materials while wall slip is present leads to an apparent macroscopic shear rate ${\dot{\gamma }}_{a}$, which is always higher than the true local shear rate $\dot{\gamma }$ applied to the material (see Figure 2) and an apparent viscosity lower than the true viscosity of the sample [16].

According to Yoshimura and Prud’homme, it is possible to correct the apparent shear rate measuring the same material at different gap heights H and the same shear stress $\tau_{R}$ to eliminate the influence of the slip velocity $u_{s}$ (see Equations (6)–(8)). The underlying assumption is that $u_{s}$ is a function of $\tau_{R}$ only and independent of H [16]:

(6) $${\dot{\gamma }}_{R,a,1}\left(\tau_{R}\right)={\dot{\gamma }}_{R}\left(\tau_{R}\right)+\frac{2\cdot u_{s}\left(\tau_{R}\right)}{H_{1}}$$

(7) $${\dot{\gamma }}_{R,a,2}\left(\tau_{R}\right)={\dot{\gamma }}_{R}\left(\tau_{R}\right)+\frac{2\cdot u_{s}\left(\tau_{R}\right)}{H_{2}}$$

(8) $${\dot{\gamma }}_{R}\left(\tau_{R}\right)=\frac{H_{1}\cdot {\dot{\gamma }}_{R,a,1}\left(\tau_{R}\right)-H_{2}\cdot {\dot{\gamma }}_{R,a,2}\left(\tau_{R}\right)}{H_{1}-H_{2}}.$$

The viscosity can then be expressed by the following equation:

(9) $$\eta \left({\dot{\gamma }}_{R}\right)=\frac{\tau_{R}\cdot \left(H_{1}-H_{2}\right)}{H_{1}\cdot {\dot{\gamma }}_{R,a,1}\left(\tau_{R}\right)-H_{2}\cdot {\dot{\gamma }}_{R,a,2}\left(\tau_{R}\right)}.$$

## 3. Geometries

**Figure 3:**
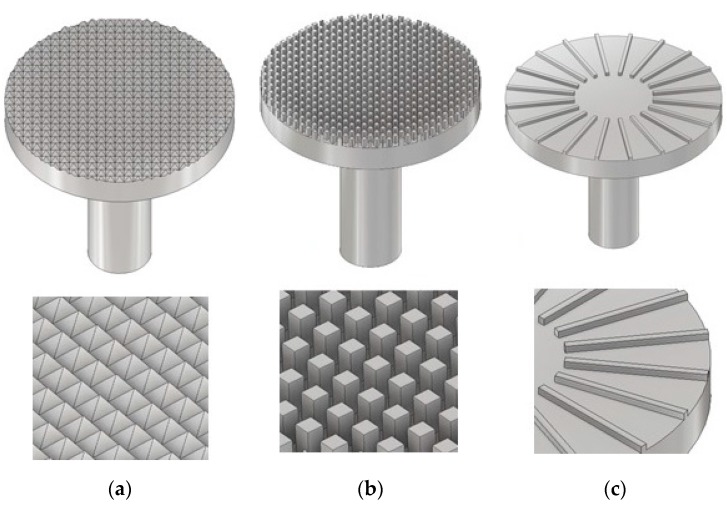
CAD construction of the modified parallel-plate systems with pyramidal structure (**a**), columnar structure (**b**), and longwise bars (**c**).

Besides the reference system (standard smooth parallel-plate (PP) system, Anton Paar PP25-TG-1), further parallel-plate systems were developed to investigate the effective gap size and its dependency on various parameters like structure size, geometry, or nominal gap. These systems include a pyramidal and columnar structure (Figure 3a,b, R=12.5 mm) as well as a structure containing longwise bars (Figure 3c, R=15 mm).

The pyramidal geometry consists of numerous uniform pyramids with a quadratic base area and an inclination of 45°. In total, four PP-systems with a pyramid height between 0.3 and 1 mm were used in this study. Since the inclination and plate diameter are fixed, the number of pyramids decreases with their height. The columnar structure was developed in accordance to a PP-system presented by Nickerson et al. [18]. It is characterized by numerous uniform and regularly arranged columns with a quadratic base area of 0.25 mm^2^. The structure depth was varied between 0.1 and 1.0 mm, and the column distance between 0.5 and 1.2 mm, resulting in seven different columnar systems. Another measuring geometry consists of 0.5 mm thick, radially arranged, longwise bars, in the following only referred to as bars. The structure depth was varied between 0.3 and 1.0 mm, and the number of bars was incremented from 16 to 20. Because of manufacturing restrictions, the plate diameter of all seven bar systems was 30 mm. The geometric dimensions for all systems are listed in the following tables (Table 1, Table 2 and Table 3). All plates were manufactured using a turning lathe and by precisely milling the structures into the plates.

## 4. Methods

All measurements were conducted with a Physica MCR 501 rheometer from Anton Paar (Graz, Austria) at T=20 °C±0.5 °C. The maximum torque accuracy was 0.5% or 0.2 μNm, respectively. The rheometer was controlled and the data were acquired with the Anton Paar software “RheoPlus” (RHEOPLUS/32 V3.31).

In order to obtain reproducible and comparable results, all measurements conducted followed the same measurement preparation and procedure, which will be described in the following. Furthermore, every measurement has been repeated at least three times. The exact compliance with a protocol is essential while measuring complex fluids with roughened plates.

### 4.1. Zero Gap Determination

After changing the measurement geometry, the zero gap was determined. In the case of smooth plates, the contact point of the plate surfaces was set as the zero gap. The approaching velocity was extremely low to reduce the influence of the zero gap error. For structured geometries, the zero gap was determined as the tip-to-tip distance.

### 4.2. Measurement Preparation

Following, the plates were lifted to a position that enabled the sample application. The Newtonian fluids and xanthan gum solutions were applied using disposable pipettes. The suspension was applied using a spatula to put as little strain as possible on the suspension, which should inhibit separation effects. Especially, the squeeze flow would cause a particle alignment that influenced the measurement. For structure depths of 1 mm, it was necessary to use a filling aid consisting of two semicircles enclosing the measuring plates to prevent the samples from leaking.

After applying the samples, the gap was set to the trimming gap 25 μm above the measuring gap. The exceeding material was trimmed in this position, and the measuring gap was set afterwards.

The approaching velocity for setting the trimming and measuring gap was 8000 μm/s in the case of Newtonian fluids and xanthan gum solutions. In preparing the suspensions, this velocity had to be reduced to 25 μm/s to stress the suspensions as little as possible and to inhibit separation effects. Again, this procedure was inevitable for reproducible results.

### 4.3. Measurement Profile

The measurement profile consisted of two sections. First, the samples were pre-sheared for t=10 s with a shear rate of $\dot{\gamma }=1\;s^{-1}$ for homogenization. Second, 30 logarithmically distributed measurement points between $\dot{\gamma }=1\;s^{-1}$ and $\dot{\gamma }=50\;s^{-1}$ were recorded. Since previous investigations showed an increasing relative error for low shear rates, a deeper analysis for $\dot{\gamma }<1\;s^{-1}$ has not been conducted. Furthermore, shear rates above $\dot{\gamma }=50\;s^{-1}$ resulted in measurement errors such as gap drainage and could not be performed. For higher-concentrated suspensions, the limit was $\dot{\gamma }=10\;s^{-1}$ because of the viscoelastic rupture of the continuous media. Therefore, in the following sections all comparisons are carried out at $\dot{\gamma }=5\;s^{-1}$, since it was part of every investigation.

Regarding the measurement values, the viscosity measured with structured plates is always lower than the true viscosity. This is due to the effective gap being bigger than the nominal gap, since the flow does not stop at the tip of the structures. Using the effective gap extension $\delta =2\cdot \delta^{\prime }$ ($\delta^{\prime }$ at the upper and lower plate), this deviation in viscosity can be analysed and corrected. However, it is worth to mention that this value is more considered to be a correction factor for the gap rather than a true penetration length of the flow into the structure, since the relation is very complex involving the shear rate as well as the shear stress. Nevertheless, this value may be used as an approximation for the maximum penetration length of the fluid flow into different geometries. To estimate δ, the measurements conducted with the structured plates were compared to measurements with commercial plane parallel plates. The viscosities measured with the commercial systems (see Section 3) will be referred to as “true viscosity” in the following discussion.

## 5. Results and Discussion

In this section the variations resulting from structured systems will be shown for all materials and similarities, and differences will be pointed out. The results presented are only exemplary. All obtained results are given in the Supplementary Materials.

### 5.1. Newtonian Fluids

The investigation of Newtonian fluids is meant to show the general influence of the structured geometries and can be used to calibrate the systems even for complex fluids, as it will be shown later. As simple Newtonian fluids, two different silicon oils Wacker AK 5000 (η=4.989 Pa·s±0.011 Pa·s) and AK 12,500 (η=12.414 Pa·s±0.043 Pa·s) were analysed. Both fluids showed a nice Newtonian behaviour in the investigated range of shear rates.

#### 5.1.1. Influence of Measuring Gap and Material

In the case of plane parallel plates, the measurement results were independent of the used gap height, if the zero gap error can be neglected [25,26]. However, for structured plates the penetration depth, and therefore δ, might change with H. To investigate this dependency, both Newtonian silicon oils, AK 5000 and AK 12,500, were measured at three different gaps—1.0, 1.2 and 1.4 mm—with all modified parallel-plate geometries. The gap heights were chosen to be independent of the zero gap error. For better comparison, the viscosity was normalised to the true viscosity:

(10) $$\eta_{norm}=\frac{\eta_{a}}{\eta }.$$

**Figure 4:**
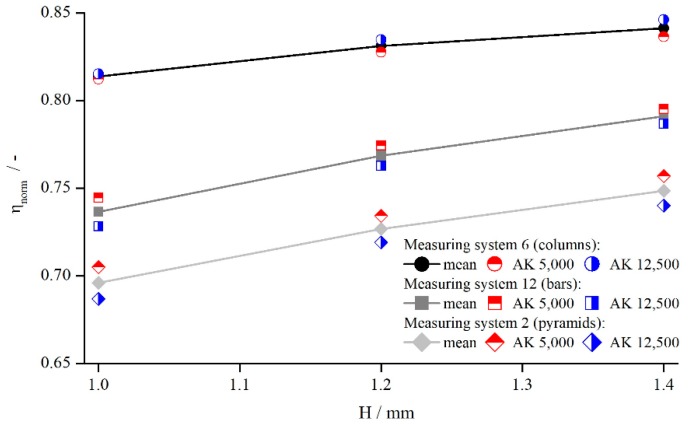
Comparison between apparent shear rate ${\dot{\gamma }}_{a}$ and true shear rate $\dot{\gamma }$ in the velocity profile between two parallel plates showing wall slip, measured with columns (system 6), bars (system 12), and pyramids (system 2).

In Figure 4, exemplary results are illustrated for pyramids (system 2), columns (system 6), and bars (system 12) at a shear rate of $\dot{\gamma }=5\;s^{-1}$. The tendency is identical for other shear rates and structures.

Figure 4 shows that the viscosity was influenced by both the structure and the measuring gap. As $\eta_{norm}$ seemed to develop linearly with a slightly degressive touch, with respect to the gap height, for all structures and viscosities, it could be explained by an effective gap extension δ, which is independent of H. Thus, for smaller gap heights, δ has a greater relative influence on the effective gap than for larger gap heights. For the different viscosities, no distinct trend can be revealed, since the results of AK 5000 were above the values of AK 12,500 in the case of bars and pyramids and vice versa for the columns. Considering all measured values (not shown for clarity purposes), the differences between AK 5000 and AK 12,500 seem to result from random errors, and there was no systematic influence of fluid viscosity on measured viscosity using structured plates. Both findings coincide with the results of Nickerson et al. [18]. However, as shown later, δ is not independent of the gap height, which appears now to be a misinterpretation due to the prior lack of further data.

#### 5.1.2. Influence of Shear Rate

**Figure 5:**
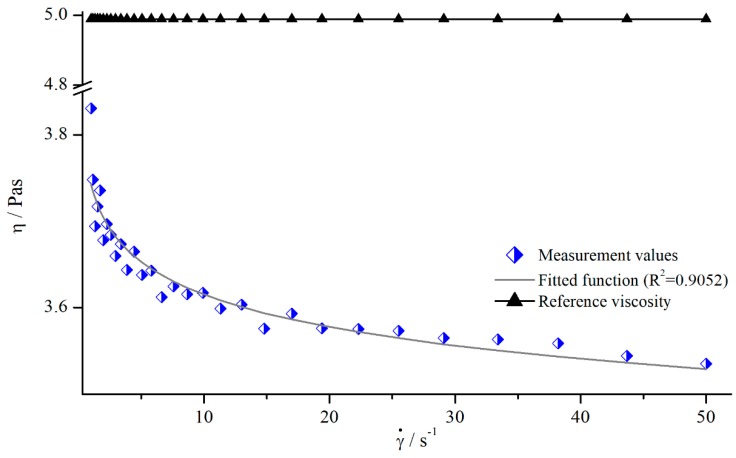
Apparent viscosity of silicon oil AK 5000 as a function of shear rate using bars (system 13) at a gap height of H=1.2 mm and reference viscosity determined with smooth plates.

In order to analyse the influence of shear rate on the viscosity values obtained by structured geometries, both silicon oils were measured with all modified parallel-plate systems. All systems showed the same qualitative behaviour (see Figure 5).

As it can be seen, the apparent viscosity decreased with increasing shear rate, approximately following a power function. This contradicts the expectation, since the used silicon oils should have Newtonian behaviour, resulting in a constant viscosity. However, this behaviour results from a deeper penetration depth leading to higher δ. Therefore, the use of structured geometries not only leads to false viscosity values, but also to the determination of a false flow behaviour.

#### 5.1.3. Determination of Gap Extension δ

The previous results revealed that the determination of a fixed correction value for δ is not appropriate, since it seems to be a function of shear rate. Additionally, from the results shown in Figure 4, it could not definitely be excluded that δ depends on the gap height. Thus, we calculated δ for different shear rates and gap heights according to following equation and fitted a correction function:

(11) $$\frac{\eta_{a}}{\eta }=\frac{H}{H+\delta }.$$

With the true viscosity η, the apparent viscosity $\eta_{a}$ was measured with the modified plates and the nominal gap height H. To derive a continual correction function, $\eta_{a}$ was approximated with a power function (see Figure 5).

**Figure 6:**
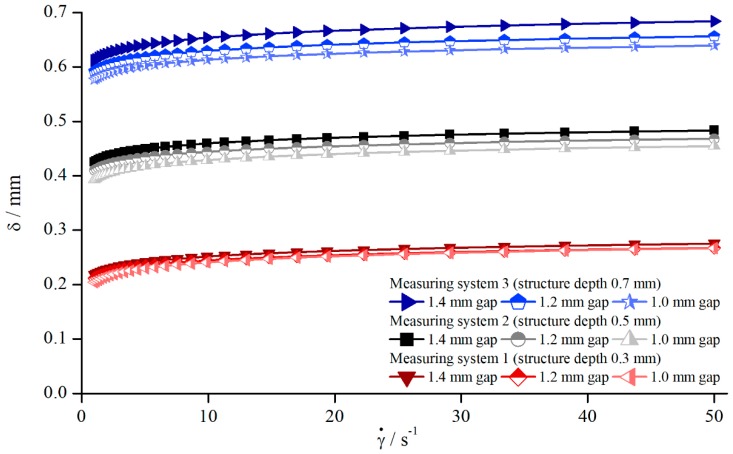
Effective gap extension δ dependent on shear rate for AK 5000, measured with different pyramidal structures (systems 1–3).

Applying Equation (11), all geometries showed the same relationship between δ, shear rate, and measuring gap so that Figure 6 shows exemplary results for pyramidal structures (systems 1–3).

Since $\eta_{a}$ was approximated with a power function, and η along with H are constant, δ follows a power function for all systems and gaps as well. Therefore, the correction function can be defined as follows:

(12) $$\delta =a\cdot {\dot{\gamma }}^{b}.$$

In contrast to the previous assumption that δ=const., it now can be clearly seen that δ is dependent on the gap height. However, compared to the influence of the structure depths, this effect appears to be rather small and can be overlooked easily. These findings oppose the prevailing opinion in the literature [18].

A possible explanation for the increase of δ with increasing gap can be found in the angular velocity. In order to produce the same shear rate at different gaps, the angular velocity in the case of greater gaps needs to be higher. Therefore, at higher velocities the flow can penetrate deeper into the structure. Since the angular velocity is directly related to inertia, this effect might be a reason for the increasing values of δ. This explanation also corresponds quite well with the decreasing viscosity shown in Figure 5, where δ was assumed to increase with increasing shear rate. Nevertheless, the relative proportion of δ decreased with increasing gap height, although the absolute value increased. This becomes obvious through Figure 4, where the normalised viscosity increased for larger gaps.

#### 5.1.4. Correction of Viscosity

**Figure 7:**
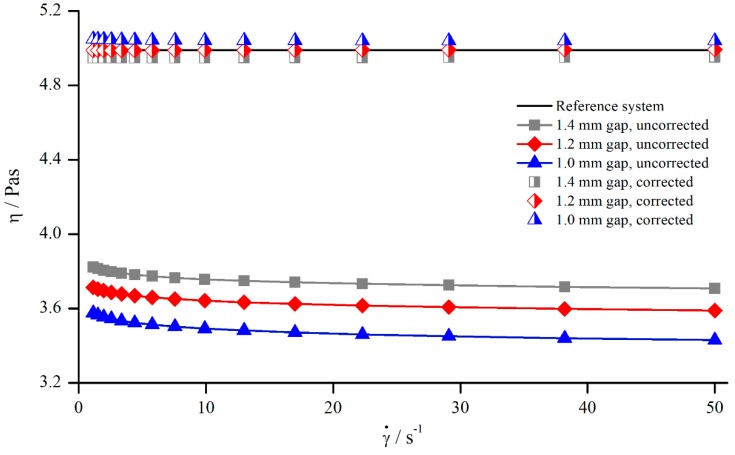
Uncorrected, corrected, and reference viscosity from silicon oil AK 5000, measured with pyramids (system 2).

In principle, it is desirable to find a gap-independent correction function. As seen before, in investigating Newtonian fluids, δ varies little for different gaps (Figure 6), which allows for the development of a correction function $\delta =f\left(\dot{\gamma }\right)$ by averaging $\delta \left(\dot{\gamma }\right)$ over the gap height. The averaged values were then approximated using a power function. Figure 7 shows the apparent and corrected viscosity following this method.

Although the gap-dependency of δ was neglected while developing the correction function, Figure 7 demonstrates that the correction of the apparent viscosity was possible with this general approach. The uncorrected values differed by 3.56% and the corrected values only by 0.97%.

**Figure 8:**
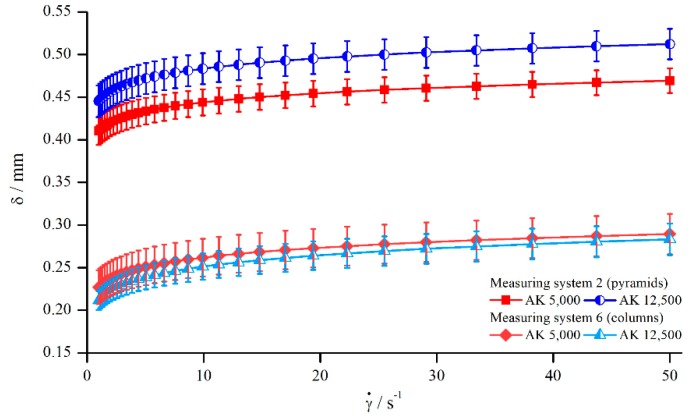
Correction function for the silicon oils AK 5000 and AK 12,500, measured with pyramids (systems 2) and columns (system 6).

However, the results presented at the beginning of this section showed that the relative influence of δ seems to be independent of the material. Thus, the correction function for both silicon oils should be comparable. To analyse this relation, the correction functions for both silicon oils measured with pyramids (system 2) and columns (system 6) are shown in Figure 8. The trend of random deviation seen before (Figure 4) also applies here. As Figure 8 shows, the correction function of the two oils measured with pyramids (system 2) differed more than it did for columns (system 6). Since the oil AK 5000 was beneath AK 12,500 for the pyramids, and vice versa for the columns, the assumption is strengthened that these deviations resulted from random errors occurring during the measurements. It has to be mentioned that this random behaviour was observed for all measurements, and the results of Figure 8 were chosen as an example of high deviations.

**Figure 9:**
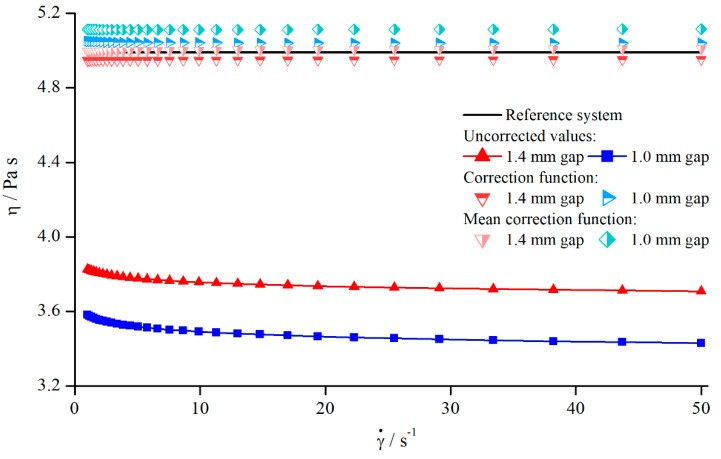
Uncorrected, corrected, and mean correction functions of AK 5000 viscosities, measured with pyramids (system 2).

In order to develop a material-independent correction function, the two correction functions of both silicon oils were averaged for each system and will be called mean correction function from here on. The results are illustrated in Figure 9.

Comparing both correction functions with regard to Figure 8, it becomes clear that the mean correction leads to viscosity values that deviate a little bit more from the reference values than the values derived by the material-dependent correction. Nevertheless, mean correction should be generally preferred when more data of Newtonian oils are available, since this correction method does not only minimize the occurrence of random errors but is also material-independent.

#### 5.1.5. Influence of Different Structural Features

**Figure 10:**
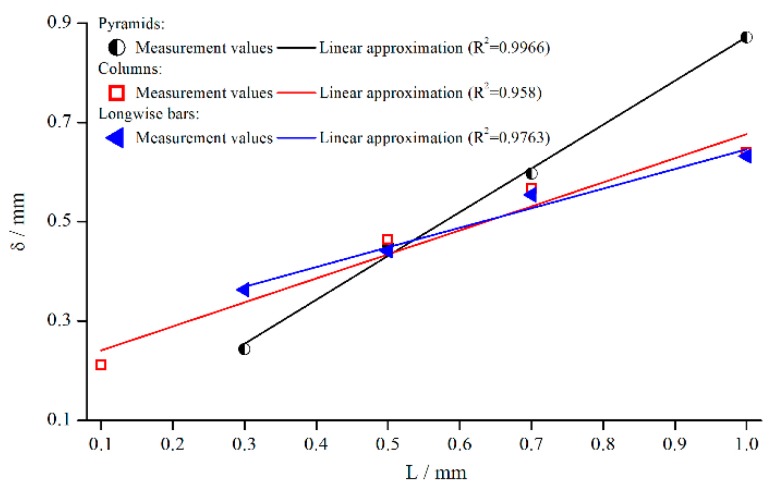
Effective gap extension δ (gap- and material-averaged) dependent on structure depth, measured with pyramidal structure (system 1–4), columnar structure (systems 5, 8, 10, 11) and bars (system 12–15) for $\dot{\gamma }=5{\;s}^{-1}$.

For the development of a general correction function, it is crucial to identify the influence of structural characteristics. Based upon the results presented earlier, the structure depth for pyramids, columns, and bars was varied at $\dot{\gamma }=5\;s^{-1}$. This not only allows for a deeper analysis of the individual structures, but also for a comparison of different structures at the same structure depth. Figure 10 shows the change of δ for all three measuring systems depending on the structure depth.

The emerging trend was similar for all structures, and δ seemed to increase linearly with the structure depth L for pyramids and bars. For columns, the linear trend was not quite as pronounced as for the other systems. However, this behaviour is somehow surprising, since other groups postulate a fixed penetration depth. A reason for the more or less linear behaviour might be that the flow can only be inhibited up to a certain distance from the valley of the structure. It is necessary to point out again that δ is only a virtual correction factor.

Furthermore, the slope of the pyramidal structure values was higher. A possible reason for that could be the decrease in the number of elements with increasing structure height in case of the pyramids (see Section 3). Therefore, it can be assumed that the fluid flow penetrated the structure even better with a smaller number of elements.

**Figure 11:**
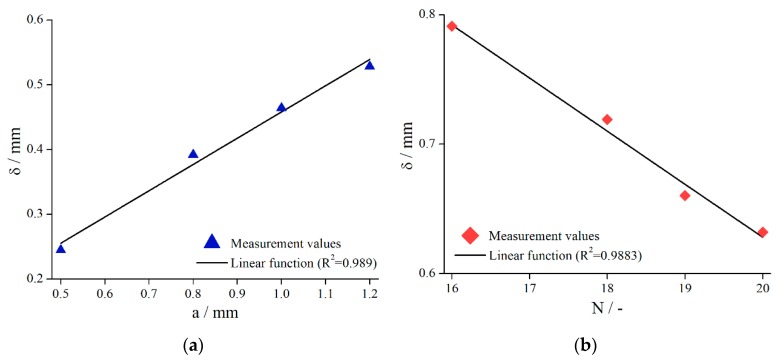
Effective gap extension δ (gap- and material-averaged) dependent on column distance (**a**) (systems 6–9, L=0.5 mm) and number of bars (**b**) (systems 15–18, L=1.0 mm) for $\dot{\gamma }=5{\;s}^{-1}$.

To strengthen this finding, the column structure (systems 6–9) and the bars (systems 15–18) have been investigated in more detail, see Figure 11.

It is obvious for both structures that, with decreasing structural elements, δ increased. Again, a linear relationship was found ($R^{2}=0.989$/columns, $R^{2}=0.988$/bars).

A possible explanation for the observed dependency of δ is that the fluid flow into the structure is less disturbed if fewer structural elements are present. Since the fluid flow can penetrate the structure even better in this case, a higher value of δ is the result.

#### 5.1.6. Comparison to Previous Studies

A summary of the following results for Newtonian fluids could be obtained:

- The relative influence of different modified geometries is viscosity-independent for the investigated range;
- A reduction of the measuring gap decreases the normalised viscosity due to the increasing influence of δ;
- The values for δ increase with increasing shear rate and gap, resulting from increasing angular velocity;
- Since the dependency of δ from the measuring gap and from the material is small, the development of a gap- and material-independent correction function $\delta =f\left(\dot{\gamma }\right)$ is reasonable;
- An increasing structure depth as well as a decreasing number of structure elements lead to increasing values of δ.

Comparing these results with previous studies, some aspects are important to be pointed out. In the study on a cleated geometry conducted by Nickerson et al. [18], they found that the normalised viscosity obtained with modified geometries was independent of the used Newtonian oil, which coincides well with the presented results. However, Nickerson et al. also showed that the viscosities measured with different cleat lengths $L_{c}$, a variable equal to the structure depth, were insensitive to this parameter, meaning $\delta \neq f\left(L_{c}\right)$. However, it could be shown that, in fact, δ is a function of the structure depth. The difference is surely a result of Nickerson et al. investigating only two cleat lengths. Furthermore, Ferraris et al. [20] used a trapezoidal-like structure with a depth of 0.38 mm, which is quite similar to the pyramids (system 1). They corrected their measurements in the range of $\dot{\gamma }\approx 0.2-8.1\;s^{-1}$ with a constant value of 0.27 mm, which is in the same range as the shown corrections for measuring system 1 (δ=0.243 mm at $\dot{\gamma }=5\;s^{-1}$). Nevertheless, they neglected the shear rate dependency $\delta =f\left(\dot{\gamma }\right)$, so a direct comparison is not possible.

### 5.2. Shear Thinning Fluid

In order to analyse the influence of modified geometries on the measurement of shear thinning fluids, a solution of 1 wt. % xanthan gum in water was investigated with all structured systems (except 3, 7, 9, 10, 14, and 16). Additionally, solutions of 2 wt. % and 3 wt. % were analysed to determine the influence of different strengths of shear thinning behaviour. Since xanthan gum is a shear thinning fluid, the reference values obtained with the commercial system PP25 as well as all other values were corrected according to the Weißenberg–Rabinowitsch correction, see Section 2.

#### 5.2.1. Comparison to Newtonian Fluids

**Figure 12:**
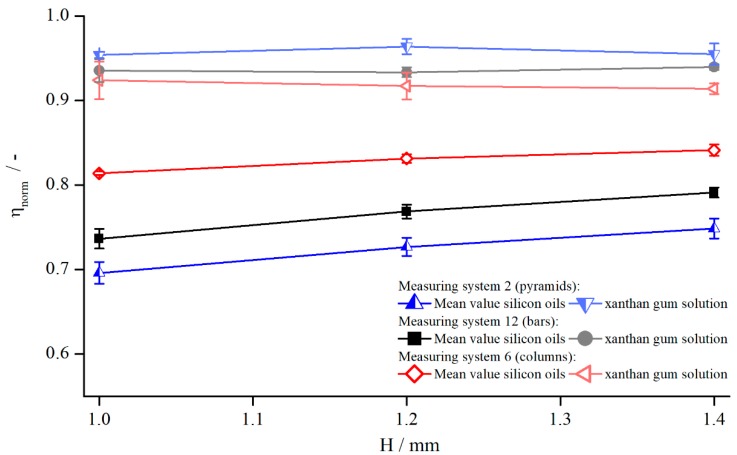
Comparison between normalised viscosities of xanthan gum solution and silicon oils dependent on gap height, measured with pyramids (system 2, L=0.5 mm), columns (system 6, L=0.5 mm), and bars (system 12, L=0.3 mm) at $\dot{\gamma }=5.05\;1/s$.

Since the flow behaviour of shear thinning fluids differs completely from that of Newtonian fluids, the influence of modified geometries might behave differently as well. Therefore, the normalised viscosity of a 1 wt. % xanthan gum solution was compared to the mean values of both silicon oils AK 5000 and AK 12,500 (Figure 12).

By comparing the normalised viscosities for both flow behaviours, it becomes clear that the influence of the structured geometries in the case of xanthan gum was not as strong as for the Newtonian oils. While the values for the silicon oils deviated in a broad range, the xanthan gum solution was quite close to the true viscosity. Nevertheless, the increase of the effective gap can be observed for xanthan gum as well.

A possible explanation for the narrow range close to the true viscosity can be found in the shear thinning behaviour of xanthan gum. In the case of structured geometries, the velocity of the upper plate was induced at the structure tips and over the complete structure depth. Therefore, the flow field entered the structure in the upper regions (tips), but the lower parts of the structure (valleys) remained unsheared. This leads to an overall lower “mean” shear rate compared to the reference system and, therefore, due to the shear thinning properties of xanthan gum, to a higher viscosity. Since this seems to counteract the increasing effective gap size, its influence decreases, leading to smaller deviations from the true viscosity.

#### 5.2.2. Influence of Measuring Gap and Material

**Figure 13:**
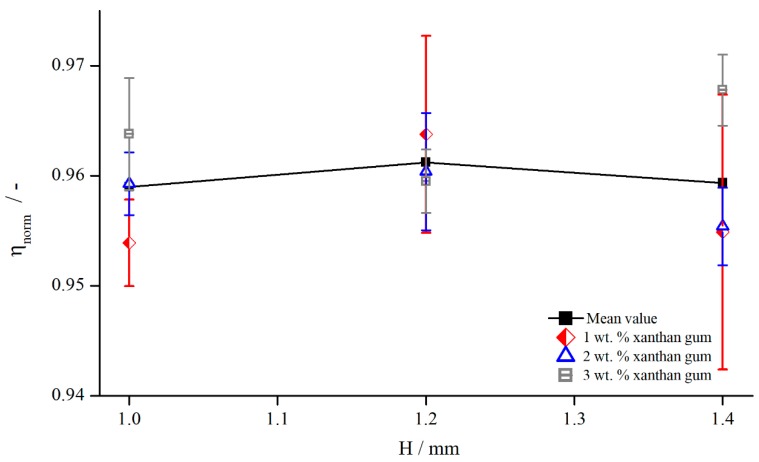
Comparison between apparent shear rate ${\dot{\gamma }}_{a}$ and true shear rate $\dot{\gamma }$ in the velocity profile between two parallel plates showing wall slip, measured with pyramids (system 2) at $\dot{\gamma }=5.05\;1/s$.

The dependency of measuring gap and the influence of shear thinning properties were analysed by measuring the normalised viscosity for three xanthan gum concentrations, 1 wt. %, 2 wt. %, and 3 wt. %, at three gap heights, 1.0, 1.2, and 1.4 mm (Figure 13).

The shear thinning properties had no influence while investigating with structured geometries. $\eta_{norm}$ varied within 1% over the different xanthan gum concentrations, which is lower than the measurement accuracy. Therefore, the assumption is reasonable that, for the shear thinning fluids used in this investigation, the effective gap is independent of shear thinning properties. However, for a decreasing extent of shear thinning properties, the normalised viscosity needs to approach the values obtained for Newtonian fluids, see Figure 12. Since these values deviated strongly from the values obtained for xanthan gum solutions, a transitional regime, or rather a critical shear thinning extent, has to exist, above which the effective gap is independent of shear thinning properties. This complex relation is out of the scope of this paper, so a detailed description is not considered.

Additionally, Figure 13 reveals the independency of the viscosity, and therefore of the effective gap, from the measuring gap. This is contrary to the results obtained for Newtonian fluids. One explanation might be that the already mentioned effect of higher viscosity due to the lower shear rates developed in the same way as the gap height and countered this effect (compare Figure 12). Furthermore, it could also be plausible that the influence of structured geometries on the viscosity was much lower than it was for silicon oils. This becomes also obvious through the fact that the normalised viscosities were higher and in a narrower regime than they were for silicon oils, see Figure 12. This leads to the assumption that δ indeed may change with the measuring gap, but this change is on the same order as random measurement errors; therefore, no relation between normalised viscosity and measuring gap can be observed.

#### 5.2.3. Influence of Shear Rate

**Figure 14:**
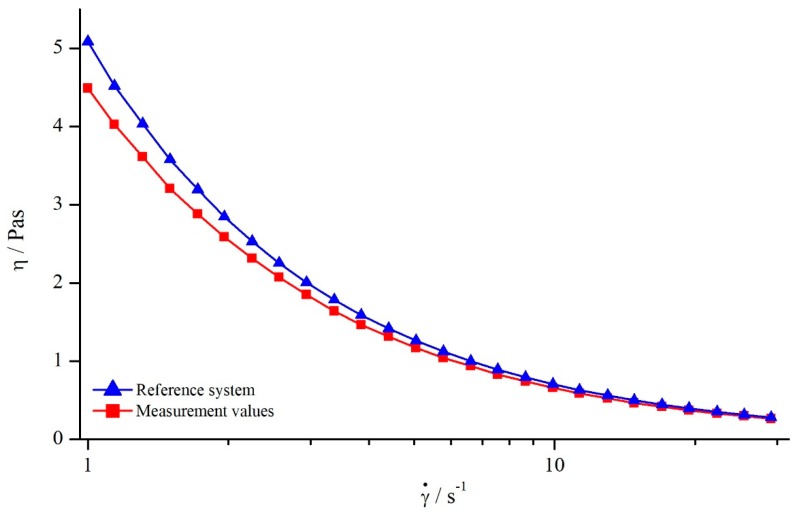
Comparison between viscosity values of 1 wt. % xanthan gum solution obtained for columns (system 6) and reference system at H=1 mm.

Analogous to the investigation on silicon oils, the influence of shear rate on the apparent viscosity was determined. To this end, the viscosity of a 1 wt. % xanthan gum solution was measured over the shear rate with all systems. Since all geometries showed the same relation, the exemplary results using a columnar structure (system 6) as well as the reference viscosity, both measured at H=1 mm, are illustrated in Figure 14.

Comparing the apparent viscosity derived by the structured geometry with the reference viscosity, it becomes obvious that the apparent viscosity deviated especially for low shear rates. With increasing shear rate, this deviation became smaller, leading to nearly the same values for apparent and reference viscosity. The decreasing deviation between apparent and reference viscosity leads to the assumption that δ might be decreasing with increasing shear rate as well. Therefore, additional investigations were conducted for determining δ.

#### 5.2.4. Determination of Gap Extension δ

**Figure 15:**
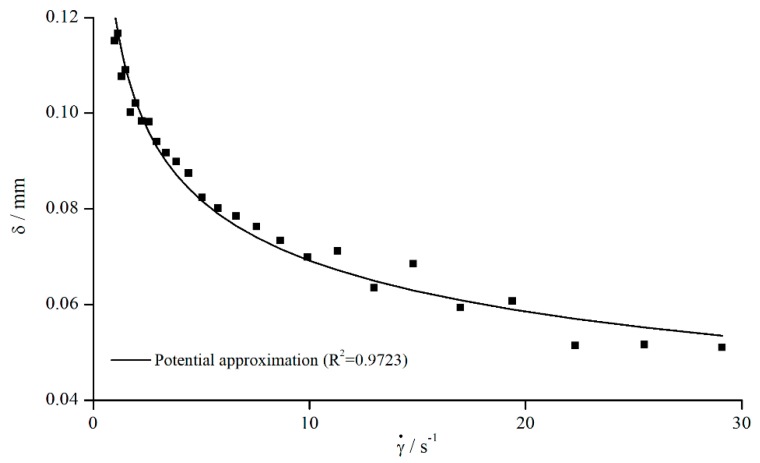
Effective gap extension δ (gap- and material-averaged) dependent on the shear rate of 1 wt. % xanthan gum solution obtained for columns (system 6, H=1 mm).

Since the xanthan gum solution showed a dependency on $\dot{\gamma }$ as well, the determination of a constant value for δ is not possible. Thus, as it was done for silicon oils, δ has been determined in the same way (compare Section 5.1, determination of gap change δ). Figure 15 shows the effective gap extension δ over the shear rate for a columnar structure (system 6) at a gap height of 1 mm.

As Figure 15 illustrates, δ decreased with increasing shear rate. Therefore, with increasing shear rate, not only the influence of viscosity on the absolute change of δ is reduced, but also the relative influence, which is contrary to the results obtained for silicon oils.

The behaviour of δ confirms the hypothesis formulated before, when the normalised viscosities for silicon oils and xanthan gum solutions were compared. As stated, the flow can penetrate into the structure when using modified geometries, leading to a lower “mean” shear rate in the gap than prescribed by the rheometer. Combined with the shear thinning properties of xanthan gum, a higher viscosity is determined, leading to smaller values of δ. This could be explained when looking at the shear rates. Since the differences between prescribed shear rate and actual induced shear rate increased with increasing shear rate, the shear thinning behaviour of xanthan gum counteracted the effect of a greater effective gap size more and more, leading to a higher viscosity; therefore, a smaller δ is determined.

#### 5.2.5. Correction of Viscosity

**Figure 16:**
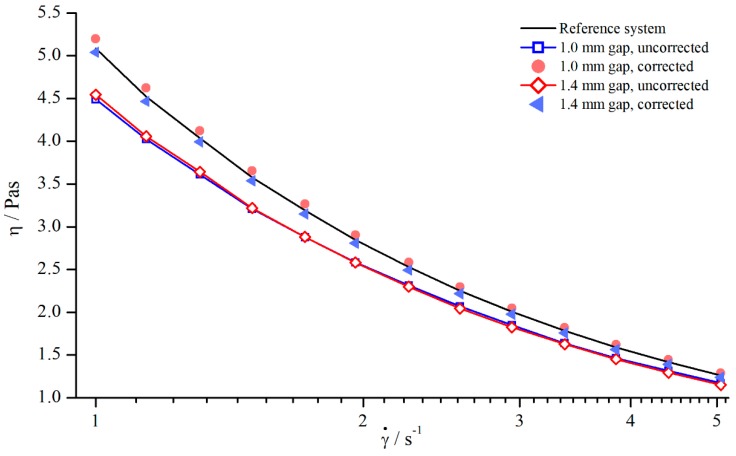
Correction of viscosity values obtained for columns (system 6) at H=1.4 mm and H=1.0 mm.

Since shear thinning fluids behave completely different compared to Newtonian fluids, the correction function cannot be transferred directly. Therefore, new correction functions have been developed following the same procedure as described before. The uncorrected values measured with columns (system 6) at two different gap heights, 1.0 mm and 1.4 mm, as well as the corrected values are shown in Figure 16. It is worthwhile to mention that, for the used xanthan gum solutions, only the gap-independent correction function has been determined.

The comparison between corrected viscosities and reference viscosity shows that the application of a gap-independent correction function $\delta =f\left(\dot{\gamma }\right)$ is possible for shear thinning fluids as well, since the corrected values deviated only slightly from the reference values.

#### 5.2.6. Influence of Different Structural Features

**Figure 17:**
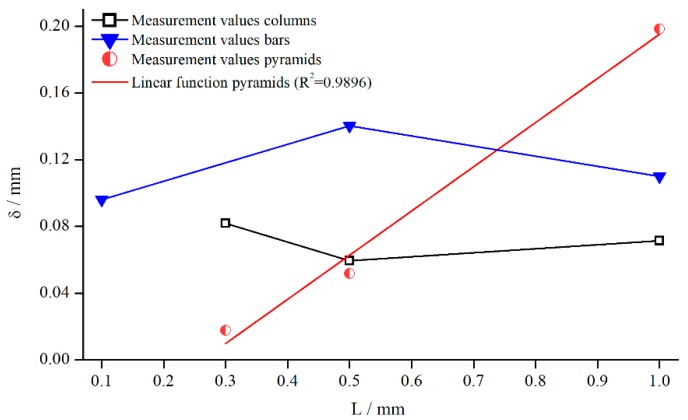
Influence of structure depth L on δ measured at $\dot{\gamma }=5.05\;1/s$.

In contrast to the Newtonian results, there seems to be no clear relationship between δ and the structure depth L, but the kind of structure is of greater importance (Figure 17). For columns and bars, δ just seemed to scatter around a fixed value over L in the range of typical measurement errors. The reason is surely the same counteracting effect that occurred at different gap heights combined with the overall lower relative influence of structured geometries on shear thinning fluids. However, in the case of pyramids, again a linear increasing δ with increasing structure depth can be observed ($R^{2}=0.9896)$. Thus, the trend that the structure depth has the strongest influence in case of pyramidal structures is confirmed. As mentioned before, this effect results from the connection of pyramid height to number of pyramids.

**Figure 18:**
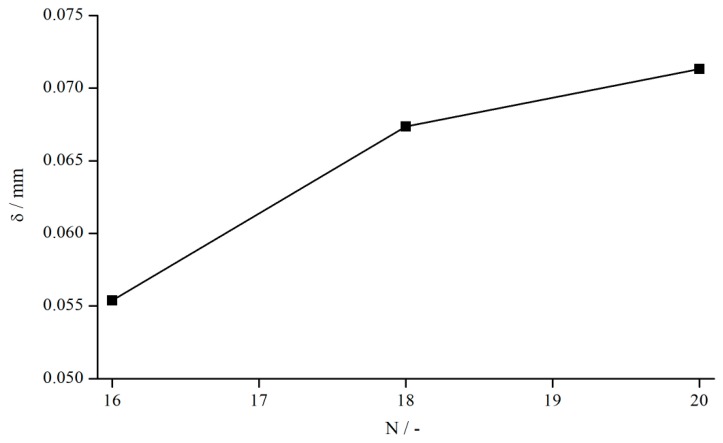
Influence of number of bars (systems 15, 17, and 18) on δ, measured at $\dot{\gamma }=5.05\;1/s$.

Since an increase in the number of structural elements showed a decrease of δ while measuring Newtonian fluids, this relation was also analysed for the 1 wt. % xanthan gum solution (Figure 18).

δ seems to rise with an increasing number of bars, which is contrary to the results observed for Newtonian oils. On an absolute scale, however, the deviation is so minute that measurement errors cannot be excluded completely. Nevertheless, also from a physical point of view, that behaviour seems reasonable. For a larger number of bars, the fluid cannot penetrate the structure as well as it can for a smaller number of bars, leading to greater difference in velocity between the fluid within the structures and in the bulk. Thus, the “mean” shear rate is higher in the case of more bars, resulting in a smaller viscosity compared to a measuring system with fewer bars and, therefore, to a bigger δ.

#### 5.2.7. Comparison to Previous Studies

The previous investigations on shear thinning fluids lead to the following results:

- The relative influence of structured geometries is far smaller than that observed for Newtonian fluids and is also independent of the strength of the shear thinning effect in the range of the investigated fluids;
- In contrast to Newtonian fluids, no exact relationship between measuring gap and normalised viscosity was observed, which is the result of two counteracting effects—gap height and shear thinning—combined with the overall small influence of structured geometries;
- The values for δ decrease with increasing shear rate, resulting from complex shear fields combined with shear thinning properties of xanthan gum solutions;
- The development of a gap-independent correction function $\delta =f\left(\dot{\gamma }\right)$ is possible, since observed deviations due to different gaps heights are small;
- Because of the relatively small changes of δ, it was not possible to reveal if δ is dependent on changes in structure depth in the case of columnar structures and bars;
- Concerning pyramids, an increase of structure depth, leading to a decrease of number of elements, leads to an increase of δ;
- An increase of bars leads to an increase of δ.

Concerning all shear thinning fluids, it is important to mention that the development of a general material-independent correction function is not possible since these fluids differ from each other regarding the strength and onset of shear thinning properties.

Comparing the results obtained for shear thinning xanthan gum solutions with the results observed for Newtonian fluids, it is obvious that δ is not only a function of measuring parameters, such as shear rate or gap height, but is also a function of the investigated material. This is contrary to the assumption made by Nickerson et al. [18], who stated that δ is independent of material properties, including complex fluids.

### 5.3. Suspensions

The suspensions investigated in this study consisted of the silicon oil AK 5000 as matrix fluid and poly(methyl methacrylate) (PMMA) particles (d=34.3±0.16 μm, Spheromers CA40, Microbeads AS, Skedsmokorset, Norway) of different concentrations. The basic idea of this study is to show the usability of structured geometries to investigate suspensions that tend to show wall slip. To this end, a 25 wt.% (21.2 vol.%) suspension was measured using all structured systems (except from systems 3, 7, 9, 10, 14, and 16). Additionally, we investigated a 5 wt.% (4.1 vol.%) and 35 wt.% (30.3 vol.%) suspension with the pyramidal structure (system 1). All reference values were obtained with the system PP25-TG-1.

In the first step, we compared the normalised viscosity and the effective gap extension δ of the 25 wt.% suspension with the values for pure silicon oil. In order to analyse the influence of different concentrations, these values were then compared to suspensions with 5 wt.% and 35 wt.% PMMA-particles. The investigation was concluded by correcting the viscosity values for the 25 wt.% suspension with regard to the already conducted correction of silicon oils.

It is worthwhile to mention that the reference values obtained for the 5 wt.% suspension were only corrected according to the Weißenberg–Rabinowitsch-correction, see Section 2, since the suspension did not exhibit any recognizable wall slip. The same applied for the values determined by the modified geometries. The other reference values (25 wt.% and 35 wt.%), on the contrary, were corrected using the wall slip correction as described by Yoshimura und Prud’homme, see Section 2, using three instead of two gap widths to obtain more precise values. Furthermore, all suspensions (5 wt.%, 25 wt. %, and 35 wt. %) showed a nearly Newtonian behaviour without the existence of yield stress.

#### 5.3.1. Comparison to Newtonian Fluids

**Figure 19:**
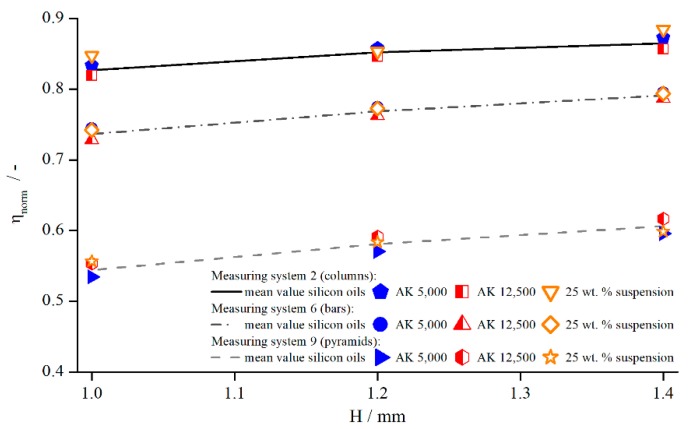
Normalised viscosities of silicon oils and 25 wt. % suspension dependent on the measuring gap, measured with columns (system 2), bars (system 6), and pyramids (system 9) at $\dot{\gamma }=5.05{\;s}^{-1}$.

The influence of adding PMMA particles to Newtonian oils on the normalised viscosity was investigated by measuring the 25 wt.% suspension with three modified geometries, with columns (system 5), bars (system 12), and pyramids (system 4), at three different gap widths (Figure 19). The viscosity values for each fluid (25 wt.% suspension, AK 5000, and AK 12,500) obtained with structured geometries were normalised to each corresponding (corrected) reference viscosity measured with the standard reference system PP25-TG-1.

Figure 19 shows that, for the same measuring system and gap width, no significant difference existed between the normalised viscosity of suspensions and pure silicon oil. The relative influence remained the same. Therefore, the assumption is reasonable that the effective gap extension δ for the suspension might not deviate from the values of the silicon oils as well. To this end, Table 4 shows the values for δ derived from the correction functions for the different materials measured at a shear rate of $\dot{\gamma }=5.05\;s^{-1}$.

It is obvious that δ was identical (within the margin of error) for suspensions and the Newtonian silicon oils.

**Figure 20:**
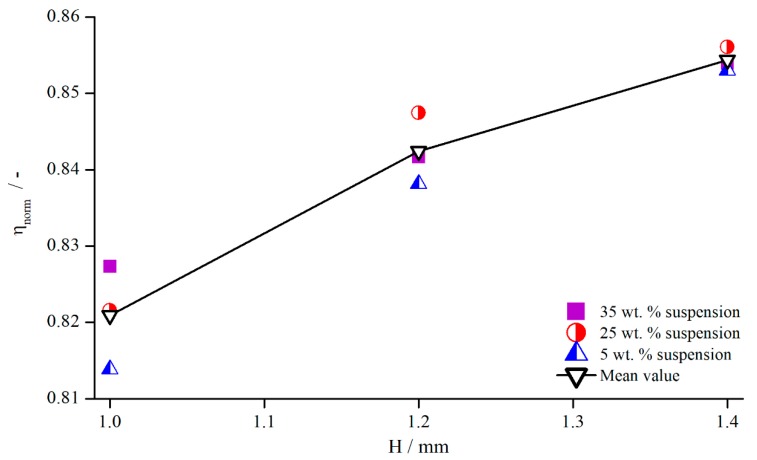
Normalised viscosities of suspensions for different concentrations, measured with pyramidal structure (system 1) at $\dot{\gamma }=5.05{\;s}^{-1}$.

In order to analyse how these values behave for different concentrations, the 5 wt.% and 35 wt.% suspensions were measured with the pyramidal structure (system 1). The normalised viscosities are illustrated in Figure 20, and the values for δ are listed in Table 5.

**Figure 21:**
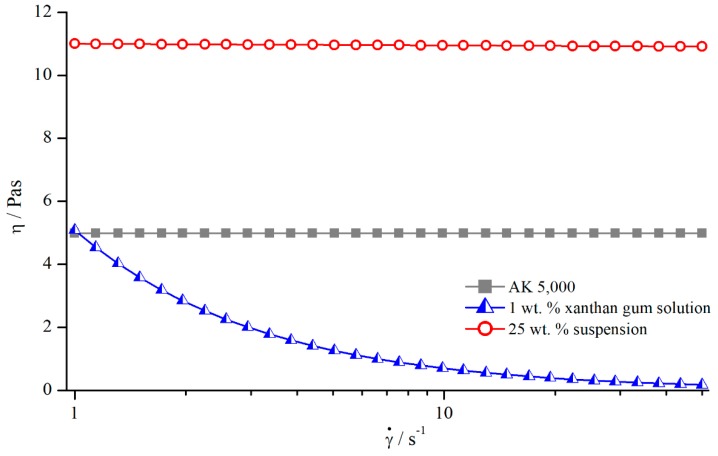
Flow curves of silicon oil AK 5000, 1 wt. % xanthan gum solution, and 25 wt. % suspension, measured with reference system.

These results show that no visible dependency between the relative influence of structured geometries and particle concentration existed, since all values deviated only slightly from each other without any apparent relation. The reason for this is surely the only weakly shear thinning flow behaviour of the suspensions with particle contents of up to 35 wt. % (see Figure 21). For clarity, only exemplary measurement values are shown for the suspension and the xanthan gum solution, but the individual flow behaviour remains the same, independent of the concentration.

The investigation of PMMA suspensions with different concentrations and the comparison to Newtonian fluids shows that the relative influence of structured geometries seemed to be the same for suspensions as well as for the Newtonian fluids used in this study. Since the flow behaviour of the suspensions was only weakly shear thinning, the assumption can be made that the relative influence mainly depends on the characteristics of the flow properties.

Concerning the influence of parameters such as shear rate on the results obtained for suspensions, it is worthwhile to mention that we observed the same dependency as for Newtonian fluids. Therefore, a closer analysis of these parameters will not follow as it already happened in Section 5.1. Nevertheless, the correction of viscosity values has been conducted, which is presented in the following section.

#### 5.3.2. Correction of Viscosity

Since the investigated suspensions and silicon oils showed a comparable flow behaviour and reaction regarding the use of structured geometries, the same correction procedure as for Newtonian oils should be applicable to suspensions. Therefore, a gap-independent correction function was calculated and applied to the suspension viscosity. However, far more important than the gap independency is the material-independent correction. Since the derivation of a material-independent correction function was possible in the case of Newtonian oils, and the suspension flow behaviour only showed a slight tendency for shear thinning, the same material-independent correction function obtained for Newtonian fluids might be applicable to the suspensions. Therefore, the suspension viscosity was not only corrected using the gap-independent correction function derived from the suspension values but also using the material-independent function derived for Newtonian oils (see Section 5, correction of viscosity).

**Figure 22:**
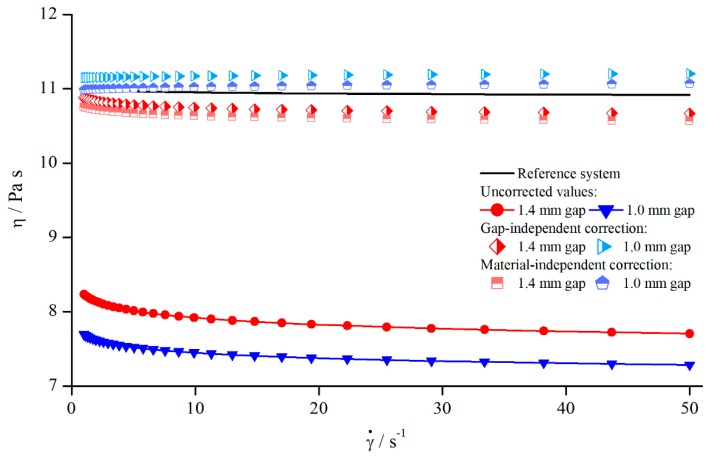
Uncorrected, corrected, and reference viscosities of 25 wt. % suspension, measured with columns (system 5).

**Figure 23:**
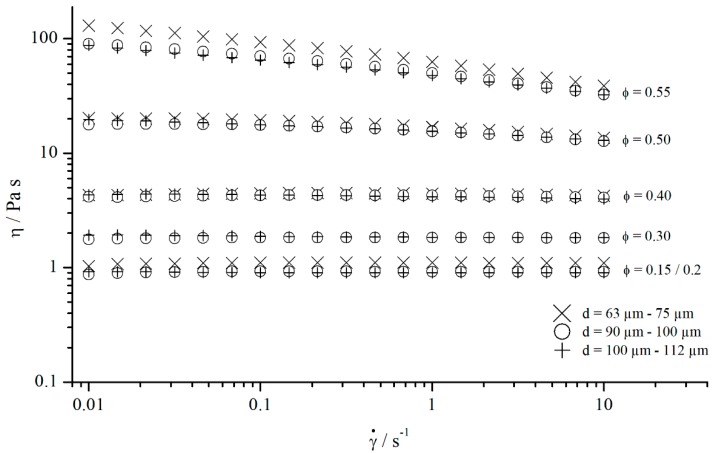
Flow curves of suspensions containing different particle volume fractions ϕ.

As expected, both methods yielded nearly the same results (Figure 22). Besides that, the apparent weakly shear thinning behaviour occurring at lower shear rates for measurements with structured plates could be corrected effectively. As shown by Jesinghausen [14] (Figure 23), even suspensions up to 50 vol.% particle content still showed fairly Newtonian behaviour. Therefore, the results presented before are extremely valuable, since it is possible to calibrate structured PP systems for suspensions up to 50 vol. % particle content using Newtonian materials.

#### 5.3.3. Comparison to Previous Studies

The results for the investigated suspensions can be summed up in the following statements:

- The normalised viscosities for the investigated concentrations, 5 wt. %, 25 wt. %, and 35 wt. %, do not deviate from the normalised viscosities obtained for silicon oils;
- The normalised viscosity of suspensions shows the same gap dependency as silicon oils;
- For the investigated suspensions, no significant deviation in the effective gap extension δ from silicon oils could be observed;
- Since the suspensions behaved only in weakly shear thinning manner, the assumption can be made that the relative influence of structured geometries mainly depends on the flow properties of the investigated materials;
- The viscosity can be corrected using a gap-independent correction function;
- Since the flow behaviour of the suspensions was only weakly shear thinning, the material-independent correction function derived for Newtonian oils could be applied to correct the suspension viscosity values, showing that slight deviations from Newtonian flow behaviour do not pose a problem.

As described before, the results obtained for silicon oils and suspensions show approximately the same results concerning the effective gap extension δ. This corresponds with the results derived by Carotenuto et al. [21], who observed that a suspension made of a Newtonian fluid and hollow glass beads (35 vol. %) flows within a porous medium (sandpaper) exactly as the single-phase Newtonian fluid. However, this relation is surely no longer given if the suspensions show a yield stress. Considering the flow curves shown in Figure 23, it can be assumed that the development of yield stress only occurs at particle contents >55 vol.% for suspensions with round and uncharged particles.

## 6. Conclusions

The use of structured geometries is a common way to prevent apparent wall slip while measuring wall slip-prone materials such as suspensions. The huge advantage of those geometries is the significantly lower measuring effort, since wall slip correction methods require measurements at two or more gaps. In this paper, we have demonstrated that the application of these geometries results in a higher effective gap size, which leads to the determination of incorrect viscosity values. However, the correction of viscosity is possible by adding the effective gap extension δ to the prescribed gap height H. For Newtonian fluids, we were able to develop a gap- and material-independent correction function $\delta =f\left(\dot{\gamma }\right)$. Since the xanthan gum solutions showed a strongly shear thinning flow behaviour, the results were incomparable to Newtonian fluids; therefore, the Newtonian correction functions could not be applied. On the contrary, the suspension only showed a weakly shear thinning flow behaviour, so the material-independent correction function derived for Newtonian fluids could be applied successfully to the suspension. This is a quite valuable result, since the structured systems can be used for suspensions up to 50 vol. % particle content after being calibrated for Newtonian oils.

## Figures and Tables

**Table 1:** Geometric dimensions of the pyramidal structure, R=12.5 mm.

Measuring System No.	Structure Depth (Pyramid Height) L/mm	Side Length of Base Area b/mm
1	0.3	0.6
2	0.5	1.0
3	0.7	1.4
4	1.0	2.0

**Table 2:** Geometric dimensions of the columnar structure, R=12.5 mm.

Measuring System No.	Structure Depth (Column Height) L/mm	Distance Between Columns a/mm
5	0.1	1.0
6	0.5	0.5
7	0.5	0.8
8	0.5	1.0
9	0.5	1.2
10	0.7	1.0
11	1.0	1.0

**Table 3:** Geometric dimensions of the bars, R=15 mm.

Measuring System No.	Structure Depth (Bar Height) L/mm	Number of Bars N/−
12	0.3	20
13	0.5	20
14	0.7	20
15	1.0	20
16	1.0	19
17	1.0	18
18	1.0	16

**Table 4:** Values for δ for different measuring systems at $\dot{\gamma }=5.05{\;s}^{-1}$.

Measuring System	Effective Gap Extension δ/mm		
	Silicon Oil AK 5000	Silicon Oil AK 12,500	25 wt.% Suspension (AK 5000)
1 (pyramids)	0.23±0.006	0.25±0.004	0.22±0.011
4 (pyramids)	0.91±0.040	0.83±0.032	0.86±0.071
5 (pyramids)	0.44±0.023	0.49±0.016	0.48±0.029
8 (columns)	0.20±0.003	0.22±0.009	0.19±0.014
12 (bars)	0.35±0.009	0.37±0.004	0.35±0.009
17 (bars)	0.73±0.068	0.71±0.072	0.70±0.089

**Table 5:** Values for δ for suspensions of different concentrations, measured with pyramidal structure (system 1) at $\dot{\gamma }=5.05{\;s}^{-1}$.

AK 5000	AK 12,500	5 wt.%	25 wt.%	35 wt.%
0.23±0.006	0.25±0.004	0.23±0.007	0.22±0.011	0.22±0.015

## Supplementary Materials

The following are available online at https://www.mdpi.com/1996-1944/13/2/467/s1, all measuring data is provided in the supplementary materials.
