# Supplementary material for: Absolute Rheological Measurements of Model Suspensions: Influence and Correction of Wall Slip Prevention Measures
Source: Materials (Basel). 2020 Jan 18;13(2):467. doi: 10.3390/ma13020467 (PMC7013820; doi:10.3390/ma13020467)
Supplement: Supplementary file 1 [file materials-13-00467-s001.zip › Explanation excel tabs.pdf]

Explanation Excel Tabs

For a better overview in the paper, the original numbers of the measuring geometries, as used for the evaluation in the excel-sheets of the supplementary material, were changed. Therefore, the following descriptions and the table should be used to understand the excel tabs and to assign the tab numbers to the numbers of the measuring geometries used in the paper.

- Ergebnisse = overview of the results
- V = template for evaluation
- Numbers = number of measuring geometries (must be converted, see following table)

**Tab number**	**Number of measuring geometry in paper**
1	Standard PP25
2	11
3	Not evaluated
4	6
5	8
6	5
7	1
8	2
9	4
10	15
11	18
12	17
13	12
14	13
15	Not evaluated
16	3
17	14
18	16
19	7
20	9
21	10
